# Burnout syndrome in different teaching levels during the covid-19 pandemic in Brazil

**DOI:** 10.1186/s12889-023-15134-8

**Published:** 2023-02-03

**Authors:** Daniela Karine Ramos, Bruna Santana Anastácio, Gleice Assunção da Silva, Leila Urioste Rosso, João Mattar

**Affiliations:** 1 Universidade Federal de Santa Catarina, Florianópolis, Brazil; 2 Federal Institute of Santa Catarina, Florianópolis, Brazil; 3 Centro Paula Souza, São Paulo, Brazil; 4 Pontifícia Universidade Católica de São Paulo, São Paulo, Brazil

**Keywords:** Basic education, Digital competence, Digital literacy, Early childhood education, Health conditions, Higher education, Stress, Teacher burnout, Teacher workload, Teaching conditions

## Abstract

**Background:**

This article’s purpose is to compare burnout syndrome indicators at different levels of teaching in Brazil during the covid-19 pandemic. The comparison also considers the teachers’ quality of life and health, working conditions, and digital competence.

**Methods:**

The hypotheses of this study are that there are statistically significant differences in teachers’ burnout rates, quality of life, working conditions, and digital competences depending on the teaching level. A mixed-methods ex-post-facto survey involved 438 Brazilian teachers, with a mean age of 42.93 years (SD = 9.66), 330 females (75%) and 108 males (25%). Data were collected through an online questionnaire. Statistical analysis of variance (ANOVA) tests was performed to compare groups, the Tukey test for paired comparison of the analyzed groups, and the chi-square to verify the association between variables.

**Results:**

Higher levels of digital competence were associated with lower burnout syndrome scores. Elementary and middle school teachers presented worse quality of life and health indexes. Adapting pedagogical work involved learning but also overwork, exhaustion, and frustration.

**Conclusions:**

The study concludes that basic education teachers had higher burnout rate scores than higher education teachers during the covid-19 pandemic and that early childhood education should be treated as a separate category.

**Trial registration:**

Ethics approval was obtained from the University of Santa Catarina (UFSC) Research Ethics Committee (4.432.063, December 7, 2020). Informed consent was obtained from all subjects.

**Supplementary Information:**

The online version contains supplementary material available at 10.1186/s12889-023-15134-8.

## Background

The covid-19 pandemic resulted in social isolation imposed to prevent the spread of the virus. Thus, educational institutions had to adapt to remote emergency teaching. Factors related to the asymmetry of access to technologies, increased demand for work, and absence of face-to-face interactions, among others, contributed to a high level of emotional stress [1–8].

Burnout symptoms combined with other conditions can impact the educational system, affecting the teaching goals, educational environment, and quality of learning. These aspects can aggravate issues associated with teacher exhaustion, generating physical, emotional, and social problems, diminished work performance, and adverse results in the student’s teaching and learning processes [9]. Stress results from the incompatibility between resources and work demands; during the covid-19 pandemic, teachers assumed distance learning challenges without the appropriate training and digital resources to develop quality pedagogical activities [10–14]. Another factor to be highlighted is that the family side of the work-life equation has expanded, and the high work-home integration has disoriented the location and staging of roles related to work and family, mainly for families with young children [15], also implying gender inequality [16].

There are different functions and attributions in the school environment according to the educational level at which teachers work. Before the covid-19 pandemic, some studies researched the relationship between burnout syndrome and educational levels [17–19].

The study conducted by Li et al. [9] before the pandemic with 1795 Chinese preschool teachers showed a high burnout rate (53.2%) among participants, highlighting that 56.5% of teachers presented medium or high levels of emotional exhaustion, 35.6% medium or high levels of depersonalization, and 21.8% a low level of professional accomplishment. Furthermore, Li et al. [9] identified that preschool teachers who worked in public schools showed a higher burnout rate compared to private school teachers. Gabriel et al. [20] also pointed out differences in the effects on teachers and educational managers of a private elementary school and an elementary and middle public school in São Paulo (Brazil) during the covid-19 pandemic, involving different procedures for different realities. In addition to dealing with educational issues, the public school faced a challenging social scenario, including hunger, amplified by the action of the virus. These factors resulted in extreme social concern among teachers and managers.

The results of another survey conducted before the pandemic with 121 teachers from kindergarten to fourth grade of nine Ohio State elementary schools indicated high-stress levels in 93% of teachers surveyed, associated with the highest means of teacher burnout [21]. Additionally, the research by Flack et al. [10] conducted with 3556 teachers in schools in Australia and New Zealand at the beginning of the pandemic identified that primary school teachers were significantly more prone to stress levels as they could not communicate online effectively with students. The findings related to communication difficulties show that elementary school students had a more limited ability to communicate through technology due to family supervision. Besides that, primary teachers mention more significant concerns about the support of parents because the younger the students, the greater the need for interventions.

Pressley [22] aimed to obtain insight into the impact of covid-19 on teachers’ anxiety and exhaustion. The sample included 359 K-12 teachers in the United States. Generally, aspects such as anxiety about teaching demands, communication with parents, and administrative support were significant. The study indicated that schools should provide communication and protocols to help teachers feel safe during the pandemic. School administrators must provide educational support and guidance to alleviate anxiety, offering greater institutional support.

Shlenskaya et al. [14] aimed to evaluate the burnout of 66 Russian university professors and estimate factors that influenced their exhaustion during the pandemic. The study showed that teachers who were already integrating digital technologies and online teaching had lower levels of exhaustion and emotional stress than those who did not have this previous experience. This inference is aligned with what was observed in Oliveira’s review [23], which mentions that the increase in hours worked is often related to the difficulty in dealing with digital tools and the absence of specific training for the integration of technologies for remote teaching, a situation that has been a source of suffering for teachers, added to the fears and anxieties directly related to the context of the pandemic and the insecurity about the future.

Carlotto [24] claims that teaching, regardless of the level of education, is becoming a profession targeted by psychosocial stressors in the teacher’s working conditions. The covid-19 pandemic has brought profound changes in the relationships between teachers, students, managers, and families, with a significant increase in the teaching workload, especially for women [25], which might have increased burnout rates.

The hypothesis of this study is that there are statistically significant differences in teachers’ burnout rates, quality of life, working conditions, and digital competences depending on the teaching level. This study aims to compare teachers’ burnout syndrome indicators at different educational levels in Brazil during the covid-19 pandemic. Burnout syndrome is associated with professional activity, including factors such as emotional exhaustion, chronic stress, and depersonalization [26, 27], and teachers are one of the professional categories most affected by the syndrome [28–31].

## Methods

This study is characterized as a mixed-methods convergent design, combining quantitative and qualitative approaches and merging the data obtained to compare and discuss the results [32]. The procedures performed can be characterized as an ex-post-facto survey, as it analyzes relationships between variables that occurred in the context of the pandemic without controlling them and after the event [33]. The University of Santa Catarina (UFSC) Research Ethics Committee (4.432.063) authorized the study on December 7, 2020. Informed consent was obtained from all subjects.

### Participants

The sample was composed by convenience through disseminating of an online questionnaire on social networks and contacting professionals and educational institutions, aiming to obtain the largest number of answers. The study included 438 teachers living in Brazil’s five regions and specifically in 17 Brazilian states. Participants had a mean age of 42.93 with a standard deviation of 9.66 years.

Table 1 shows that most teachers are female and married or in a stable union at all levels of education. Except for higher education, where most teachers have master’s degrees, most have specialization degrees.

**Table 1:** Sample characterization by the educational level

Variable	ECE	ES	MS	HS	HE	Total
**Participants** (n)	42	80	75	145	96	438
**Mean Age** (SD)	40,3 (1,3)	41,4 (1,0)	43,1 (1,1)	42,9 (0,8)	45,6 (1,0)	42,93 (9,6)
**Sex**						
Female	40 (95,2%)	77 (96,3%)	59 (78,7%)	92 (63,5%)	62 (64,6%)	330
Male	2 (4,8%)	3 (3,8%)	16 (21,3%)	53 (36,6%)	34 (35,4%)	108
**Marital status**						
Single	6 (14,3%)	14 (17,5%)	21 (28%)	35 (24,1%)	21 (21,9%)	97
Married/stable union	31 (73,8%)	59 (73,8%)	48 (64%)	97 (66,9%)	70 (72,9%)	305
Divorced/separated	5 (11,9%)	5 (6,3%)	6 (8%)	13 (9%)	5 (5,2%)	34
Widower	0 (0%)	2 (2,5%)	0 (0%)	0 (0%)	0 (0%)	2
**Degree**						
High school	1 (2,4%)	0 (0%)	0 (0%)	0 (0%)	0 (0%)	1
HE incomplete	2 (4,8%)	2 (2,5%)	0 (0%)	0 (0%)	0 (0%)	4
HE complete	11 (26,2%)	25 (31,3%)	16 (21,3%)	22 (15,4%)	2 (2,3%)	76
Specialization	24 (57,1%)	43 (53,8%)	41 (54,7%)	85 (59,4%)	12 (13,6%)	205
Masters	3 (7,1%)	9 (11,3%)	18 (24%)	29 (20,3%)	38 (43,2%)	97
Doctorate	1 (2,4%)	1 (1,3%)	0 (0%)	7 (4,9%)	30 (34,1%)	39
Post-doctorate	0 (0%)	0 (0%)	0 (0%)	0 (0%)	6 (6,8%)	6

*ECE* Early Childhood Education, *ES* Elementary School, *MS* Middle School, *HS *High School, *HE *Higher Education

Most of the responding teachers work in the public sector at all levels, as shown in Table 2, with the highest percentages in middle school (89.2%) and early childhood education (88.1%).

**Table 2:** The main network of activities that teachers work by educational levels

Variable	ECE	ES	MS	HS	HE	Total
**Main working sector**						
Private	5 (11,9%)	22 (27,8%)	8 (10,8%)	17 (12,1%)	31 (33,3%)	83 (19,4%)
Public	37 (88,1%)	57 (72,2%)	66 (89,2%)	123 (87,9%)	62 (66,7%)	345 (80,6%)

*ECE* Early Childhood Education, *ES* Elementary School, *MS* Middle School, *HS* High School, *HE* Higher Education

### Data collection instruments and procedures

Data collection was based on an online questionnaire (composed of a set of objective questions and one open question) that was applied at the end of 2020. The questionnaire had the following sections:

- Profile: collected information about the personal and professional profile of the respondents, such as name, gender, age, schooling, modality of professional practice, and workload.
- Digital competence: sought to elucidate previous experiences using digital technologies in their pedagogical practices and access to digital technologies or devices. Statements included, for example, the use of email, instant communication (e.g., WhatsApp), and web conferencing platforms, where the teacher indicated whether he/her had never used it, started using it during the pandemic, or already used it. The scores were generated by adding the points corresponding to each response (0 - never used; 1 - had never used and started using during the pandemic; and 2 - already used).
- Working conditions: it sought to elucidate the conditions that included interpersonal, institutional, technical, and pedagogical support for the development of activities in remote teaching. For the verification of the support received during the pandemic period’s performance, 11 statements were systematized, such as: “My co-workers assist me in the activities of remote teaching”, which were answered through a 5-point Likert scale (I totally disagree with it … I totally agree with it).
- Quality of life and health: presented nine indicators based on the literature review (quality of sleep, number of hours of sleep, quality of food, body weight, blood pressure, humor, anxiety, gastrointestinal problems, and muscular pains), from which teachers should answer each item that expresses their conditions by selecting one of the options: “Worsened in the pandemic” (− 1), “There were no changes” (0) and “Improved in the pandemic” (+ 1), and the final number is the sum of the nine indicators.
- Maslach Burnout Inventory – General Survey (MBI): 20 statements for evaluation by the teachers related to the characteristics of burnout syndrome that were translated into Portuguese and adapted. The response alternatives used a Likert scale related to the frequency with which the respondent had the feeling or perception described, expressing themselves in 5 frequency points (never = 1 … daily = 5), and aimed to understand, for example: whether teachers felt emotionally exhausted with their work and felt that their work in remote teaching wore off. The analysis of the adaptation of the MBI considered the total score (numerical variable). Thus, the test result was analyzed as an indicator of the syndrome without having the objective of classifying or diagnosing the participants.

Several scales are used to measure burnout, such as Burnout Assessment Tool (BAT) [34, 35], Burnout Measure (BM) [36], and Copenhagen Burnout Inventory (CBI) [37]. The Maslach Burnout Inventory (MBI) [38] was chosen because there is a translation into Portuguese, and it is the most frequently mentioned and used in Brazil.

The internal consistency of the set of items that generated the indicators for burnout, working conditions, quality of life and health, and digital competences was analyzed by Cronbach’s Alpha. The results obtained were 0.83 (adapted MBI), 0.77 (working conditions), qualify of life (0.83), and digital competence (0.78). Considering the parameters that Cohen, Manion, and Morrison (2018) indicate that between 0.70 and 0.79, the instrument is reliable, and between 0.80 and 0.90 is highly reliable, these instruments might be considered reliable. Data analysis considered the effect size calculated by square Eta (Ƞ^2^). The last and open question sought to provide a space for respondents to record their reflections on their teaching performance during the pandemic, including aspects that they considered were not addressed in the questionnaire.

Three specialists, PhDs, university professors, and research experienced validated the whole instrument. The experts were invited to validate the questionnaire in a synchronous meeting. Each specialist was requested to answer the research instrument, and later, it was opened, and each question was discussed, aiming to validate the construct and content.

### Data analysis

The set of questions sought to generate scores for the dependent and independent variables of the research based on the scales used. The primary independent variable considered in the analyses was the educational level in which the teacher worked, and the dependent variables were the scores on the indicators of digital competence, working conditions, quality of life and health, and the MBI.

The software SPSS (Statistical Package for the Social Sciences) version 24 was used to perform quantitative data analysis. The data normality was investigated through the Kolmogorov and Shapiro-Wilk tests and the values of Skewness and Kurtosis. For parametric data, statistical analysis of variance (ANOVA) tests was performed to compare groups, the Tukey test for paired comparison of the analyzed groups, and the chi-square to verify the association between variables [33].

The qualitative data analysis was performed based on systematic procedures and the objectives of content description based on inference [39]. The categories and subcategories were organized according to the methodology proposed by Saldaña [40] and the support of the NVivo software to obtain greater efficacy in the treatment of results, inferences, and interpretations.

## Results

Part of the quantitative analysis involved measuring symptoms linked to burnout syndrome and was performed based on the adaptation of the MBI. Elementary and middle school teachers had the highest scores, while the lowest scores were identified in teachers working in higher education, as shown in Table 3. The quality of life and health measured by teachers’ self-assessment had consistent results that reinforce the symptoms of burnout syndrome since the worst indicators are observed in elementary and middle school teachers, and higher education teachers had the best scores compared to all other educational levels.

**Table 3:** Means and standard deviation of the score for MBI, quality of life and health, digital competence, and working conditions

Variable	ECE	ES	MS	HS	HE	F	p	es
**MBI**								
Mean	40.52	45.47	46.40	42.83	37.06	7.54	< 0.001	0.065
Standard Deviation	9.34	11.5	11.9	13.8	13.7			
**Quality of life and health**								
Mean	−5.16	−5.98	− 5.64	−4.63	−4.08	5.09	0.001	0.064
Standard Deviation	2.73	2.57	2.94	3.64	3.37			
**Digital competence**								
Mean	15.16	14.83	14.69	15.39	17.22	8.09	< 0.001	0.070
Standard Deviation	2.75	3.75	3.42	3.59	3.06			
**Working conditions**								
Mean	44.83	48.66	46.80	47.75	47.39	2.72	0.029	0.049
Standard Deviation	8.56	7.63	8.76	8.78	8.71			

*ECE* Early Childhood Education, *ES* Elementary School, *MS* Middle School, *HS* High School, *HE* Higher Education, *es* effect size

Considering that the ANOVA test indicated a significant difference in comparing the teaching levels for the analyzed variables, multiple comparisons were conducted using the Tukey posthoc test, which compares the means by combining pairs of levels based on the t-test. The MBI revealed that higher education had significantly lower scores when compared to elementary school (*p* < 0.001), middle school (*p* < 0.001), and high school (*p* = 0.005), but not early childhood education.

When comparing the scores related to the quality of life and health, except for early childhood education, the lower the educational level, the lower the quality of life and health. The analysis identified significant differences in comparisons between elementary school and higher education (*p* = 0.001), elementary school and high school (*p* = 0.022), and middle school and higher education (*p* = 0.015).

We also analyzed two other variables, digital competences and working conditions. Table 3 shows that the lowest scores related to digital competences were from teachers working in elementary and middle school, and the highest mean score obtained was by higher education teachers. The indicators of working conditions measured in the questionnaire had lower mean scores in early childhood education and higher scores in elementary and high school, followed by the score obtained in higher education, as shown in Table 3.

Comparisons between digital competence scores using Tukey’s posthoc test revealed significant differences in comparing higher education with all other levels. When comparing higher education with early childhood education, *p* = 0.010, no significant difference was found between early childhood education and any other level (*p* > 0.05). There was a significant difference only in comparing elementary and middle school to higher education (*p* < 0.001). Finally, the comparison with high school also revealed a significant difference only in the pairing with higher education (*p* < 0.001).

The analysis between education levels and working conditions in the Tukey test revealed a significant difference only when comparing early childhood education and elementary school (*p* = 0.016). Comparing the other educational levels did not identify significant differences.

Considering the qualitative results in the space for participants to register their considerations about their teaching activities during the pandemic, 210 teachers reported on their experiences, mentioning aspects not addressed in the questionnaire. The analysis of these answers followed the descriptive and emotion coding and sub-coding methods proposed by Saldaña [40], generating 14 subcategories distributed in five categories. This grouping was performed by levels of education based on the content expressed in each answer, represented in Table 4, in which the most frequent subcategories are highlighted, with the dismembering of career and technical high school level for in-depth analysis.

**Table 4:** Categories and subcategories and their percentage related to the frequency of answers by teaching levels

Categories	Subcategories	ECE	ES	MS	HS	CTHS	HE
		%	%	%	%	%	%
**Emotions**	Frustration	25	23	10	14	19	11
	Emotional fatigue	10	15	5	8	12	22
	Exhaustion	10	36	8	8	23	22
	Fear and anguish	5	5	3	6	0	0
**Need for adaptation**	Lack of face-to-face	25	13	13	14	16	11
	Resilience	0	10	3	3	5	14
	Adaptation of pedagogic work	50	31	28	31	44	38
	Boundary between work and home	0	10	3	11	12	11
**Work conditions**	Overwork	20	26	28	6	14	24
	Bureaucracy	0	3	10	3	2	5
	Training	20	3	0	0	0	0
**Students learning conditions**	Family mediation	15	5	0	0	0	0
	Lack of student participation	5	15	23	11	5	5
**Others**		20	21	13	25	7	19

*ECE* Early Childhood Education, *ES* Elementary School, *MS* Middle School, *HS* High School, *HE* Higher Education, *CTHS* (Career and Technical High School)

The most cited subcategory among teachers of all levels (except for elementary school, being the second) was the adaptation of pedagogical work, reaching 50% in early childhood education and 44% in career and technical high school. Early childhood education teachers reported challenges in a type of work that is deeply based on emotional contact with children, being the educational level where the subcategories lack of face-to-face (25%) and training (20%) were the most mentioned: “Challenging! Working with Early Childhood Education at a distance, where affection, touch, and the basis of work, in remote teaching, was and continues to be a major obstacle” (T = Teacher 80, ECE). High school teachers, for whom the adaptation of pedagogical work was the most mentioned subcategory (31%), also identified challenges: “The difficult thing was to adapt, change the work routine, family, expand (invest) in technological resources.” (T 365, HS).

Teachers who obtained 31% (elementary school) and 28% (middle school) in the adaptation of pedagogical work subcategory also reported challenges, sometimes mentioning learning in the process: “I could not dedicate myself as I should, and this made me very frustrated. But I have moved on, and I can already evaluate what has worked and what I need to rethink in my teaching practice” (T 266, ES). Career and technical high school teachers also expressed challenges: “It was good, apart from the adaptation that had to be very fast. At first it was more difficult, and in the end, I am feeling more tired.” (T 155, CTHS), although also identifying learning in this process: “I am convinced that remote teaching showed us some teaching possibilities that had not been considered before, we learned, but nothing is better than being in the classroom in person.” (T 366, CTHS).

On the other hand, higher education teachers, for whom the adaptation of the pedagogical work subcategory was also widely mentioned (38%), expressed an interesting mix of challenge and learning, or stress and fun: “In the pandemic, I felt even more sensitive to my students, we established a great relationship, and despite all the stress of the pandemic in society, we had a lot of fun. I only felt difficulty in correcting the activities, but one can adapt more.” (T 178, HE).

Lower than the frequency of the adaptation of pedagogical work, a group of three other subcategories stands out: overwork, exhaustion, and frustration. Overwork was among the most significant difficulties of middle school teachers, together with the adaptation of pedagogical work, with 28% each frequency in responses. There was also a significant frequency of this subcategory among elementary school (26%) and higher education (24%) teachers.

The replanning of classes and activities, that is, what had already been prepared for the face-to-face teaching plans had to be reconfigured, generating an extra workload: “My quality of life, the change of routine, the long hours sitting in front of a computer, the many online meetings called at the last minute; messages and delivery of activities at any time of the day, including at dawn. The various questions taken from parents and students simultaneously at any time of the day.” (T 132, ES). Over-work is expressed when teachers report exceeding office hours and receiving messages on their personal devices, including weekends, demonstrating the difficulty of imposing limits and schedules to perform their work. If, on the one hand, the use of social networks and platforms for contact with students and the institution was seen as something positive and involving much learning, on the other hand, these interactions were associated with overwork: “In remote education we start to perform a lot of bureaucratic work, taking up much more time than my workload.” (T 61, MS).

Exhaustion is directly associated with overwork. It was the most perceived subcategory among elementary school teachers (36%) and the second among career and technical high school teachers (23%). For higher education teachers, the adaptation of pedagogical work (38%), overwork (24%), and exhaustion (22%), together with emotional fatigue (22%), were the most mentioned subcategories.

Considering this was the teachers’ first experience with emergency remote teaching or even online teaching and that the pandemic was a period of restrictions and challenges, it was possible to identify many emotions in the quality data, naming it one of the categories of this study. Furthermore, for many respondents, emergency remote teaching was not experienced positively. Thus, the subcategory exhaustion was the most cited within the category emotions at many levels, having greater prominence among elementary school teachers: “I feel exhausted, demotivated, much more tired, and frustrated in thinking that many students do not have access to remote classes and many families are not participating in this process”. (T 432, ES). Frustration is strongly present in the discourse of early childhood education (25%) and elementary school teachers (23%), and the subcategory family mediation shows up only in the answers of the early childhood education teachers (15%) and elementary school teachers (5%).

## Discussion

This study aimed to compare teachers’ burnout syndrome indicators at different educational levels in Brazil during the covid-19 pandemic, also considering the following variables: teachers’ quality of life and health, working conditions, and digital competence. The hypotheses were that there are statistically significant differences in teachers’ burnout rates, quality of life, working conditions, and digital competences depending on the teaching level. The study found statistically significant differences in these four variables related to teaching levels but with specific configurations.

Before the pandemic, studies indicated high teacher burnout rates [9, 21, 28]. Remote emergency teaching involved limitations in resources, such as faculty support and training [22]. Home and work suddenly merged, teachers faced new challenges, new tools had to be incorporated into their pedagogical practice, the workload increased, and emotional and health conditions worsened. This intensified teachers’ stress, consequently affecting burnout rates [10, 13, 14, 22, 23].

The adaptation of pedagogical work was the most mentioned challenge by Brazilian teachers at all levels in the open question of this study. However, some teachers could also reflect on the learning involved in this process, with higher education teachers even mentioning fun.

Higher education teachers had the best scores in the MBI, digital competence, and quality of life and health indicators. Although higher education teachers considerably mentioned the adaptation of pedagogical work (38%), there were statistically significant differences in comparing their digital competence scores with all other levels of education. This competence may be associated with the integration of technologies before the covid-19 pandemic and is consistent with the results of the study by Shlenskaya et al. [14], which concluded that teachers who already used digital technologies and were experienced with online teaching had lower levels of emotional exhaustion compared to those who did not have this previous experience.

On the other hand, teachers working in elementary and middle school had the lowest scores related to digital competence. This is aligned with higher scores in the MBI and the worst indicators of quality of life and health. In the same direction, the study by Flack et al. [10] points out that primary school teachers were significantly more inclined to stress levels and the worst quality of life and health rates than higher education teachers.

This study identified that the lowest the quality of life scores, the higher the MBI. This corroborates the relationship between burnout symptoms and general well-being identified by Buonomo et al. [41] in Italian healthcare professionals; Vinnikov et al. [42] findings that several aspects of quality of life, such as fatigue, predicted more burnout in oncologists in Kazakhstan; and Vinnikov et al. [43] findings that health-related quality of life aspects, such as fatigue and stress were associated with burnout in rescuers in Kazakhstan. Fatigue was one of the most mentioned categories in the teachers’ comments in this study.

The study results also support the claim that the higher the indicators of digital competence, the lower the indicators related to burnout syndrome during the covid-19 pandemic, which corroborates Garcia’s [44] finding that there was a negative and significant relationship between digital competence and burnout during the covid-19 pandemic. Digital competence involves the integration of digital resources for information access and communication. The experience and the previous integration of these technologies facilitated the adaptation of the pedagogical processes from face-to-face to remote teaching. Consequently, teachers who had lesser previous educational technology experience faced a higher challenge of integrating new digital resources into their pedagogical practices.

However, early childhood education deserves a closer examination on this point. Higher education teachers had statistically significantly lower burnout indicators compared to all education levels, with an exception made for early childhood education. Also, except for early childhood education, the lower the educational level, the lower the quality of life and health scores. Furthermore, early childhood teachers had the best scores on working conditions, even when compared to higher education. At the same time, early childhood teachers were the ones that most mentioned frustration (25%), training (20%), and family mediation (15%), with the last two subcategories being also mentioned only by elementary school teachers. Besides that, early childhood teachers did not mention resilience, the boundary between work and home, and bureaucracy, while all the other levels of education mentioned these subcategories.

Different explanations can be offered for these apparently inconsistent results. First, early childhood education has objectives that markedly differentiate it from all other educational levels. Taking this into consideration, the high percentage of frustration among early childhood teachers can be explained by the fact that they had their hands tied, as care and socialization with their students were practically impossible through the available technologies, which was aggravated by the lack of training. These teachers’ work did not accomplish without family mediation; their role during the pandemic was much more to complement family and community actions — possibly the cause of lower bureaucracy and lower boundary troubles between work and home. Consequently, they faced more frustration because many parents did not contribute as expected, and as educators, they could not replace the families’ functions during the crisis. At the same time, and because of the same reasons, early childhood teachers did not feel as much pressure as other teachers regarding learning outcomes and assessment.

Another relationship identified in the results of this study was among the categories of overwork, exhaustion, and frustration. In the context of adaptation to the pandemic, it was necessary to replan classes and activities, which led to an extra workload [45]. Thus, there was a significant increase in the demands of time dedicated to professional activities. About 70% of teachers in the Flack et al. [11] study stated that planning time increased “slightly” or “significantly” and reinforced the challenge of teaching simultaneously to students in person at school and at a distance. In Oliveira’s study [23], early childhood education teachers highlighted increased working hours spent preparing for non-face-to-face classes.

In the answers to the open question, it was possible to identify emotions related to the lack of resources and training and the feelings of being helpless: “The lack of psychological support for young people in the public schools was alarming.” (T 165, HS); “We need emotional assistance to the teachers who are not valued, must somehow ad-just and condition themselves according to the demand. Who cares about us? If we are emotionally ill?” (T 73, ES). This teacher expressed his anger in a wordier way:

“Face-to-face institutions, without structure and support for remote work, associated with corporatism and power disputes in the academic field and pressures and documents from the top down without dialogue with professionals bring frustrations and have impacted even more on the illness of people who, as me, were already ill and in extreme psychic suffering by organizational practices that harm our mental health. ( …) And the professional caregivers are being cared for by whom? We take care of our students, but we were left helpless during the Pandemic by institutions, colleagues, leaders, and even cowardly pressured by laissez-faire policies of the Ministry of Education that caused only chaos to what was already chaotic.” (T 74, HE).

In addition to personal conditions, it is recognized that working conditions affected teachers’ performance during the pandemic. In the comparative study conducted by Prado-Gascó et al. [45] on the psychological impacts and risks faced by teachers from Spain and Mexico, while the coronavirus crisis was at its peak in Spain and the early stage in Mexico, teachers from both countries reported work overload during the pandemic, while at the same time, they perceived the importance of social support (defined as the availability of help from others). As noted, the teachers of this study also reported work overload and the perception of the importance of social support. The excess work characterized in the teachers’ voices is an essential component of the burnout syndrome, as well as fatigue and exhaustion, among other related expressions that had great frequency in the teachers’ statements, regardless of the educational level.

According to Pressley [22], the areas related to covid-19 are anxiety, current teaching anxiety, anxiety communicating with parents, and administrative support. Thus, schools must help teachers during an outbreak such as the covid-19 pandemic, providing guidance and support to deal with anxiety. Administrative and management support and access to the necessary tools help reduce burnout syndrome occurrences. Prado-Gascó et al. [45] consider that teachers’ perceptions about the measures taken by the responsible agencies and the perception of insufficient information and resources can influence the psychosocial risks to which these professionals are exposed, as well as burnout and possible alterations associated with it.

Brazilian legislation [46] obliges organizations to carry out entrance and exit exams, besides periodic exams on their employees every 2 years, but without the need for follow-up of specific mental aspects regarding their job and families. For example, this obligation does not include compassion at work [41]. None of the categories identified in this study qualitative analysis have a relationship with the semantic field of compassion. The Brazilian legislation was not ready to protect teachers during the de covid-19 pandemic crisis, including working conditions, burnout, and well-being. In this sense, burnout assessment tools, and the instrument used in this study, can help to identify and propose prevention programs, as noted by Borelli et al. [35].

As this study aimed to cover from early childhood to higher education, a more specific look at early childhood teachers’ context might have changed the instrument. The three professors who validated the instrument were not experts in this level of education. A teacher expressed this limitation: “I thought that many questions were not suitable for early childhood education.” (T 135, ECE).

Another limitation of this study is that it used a convenience sample, not a random one, or a stratified sample by the country’s regions, educational levels, public/private schools, gender, etc. Besides that, the sample size was less extensive because of the lack of return to the questionnaires by teachers facing many social turbulences during the pandemic.

## Conclusions

The adaptation of pedagogical work was the most mentioned category by the teachers. Furthermore, higher levels of digital competence were associated with lower burnout syndrome scores and higher quality of life and health indicators. The need to adapt the teaching work without previous experiences with integrating digital technologies into pedagogical practices contributed to overwork, frequently mentioned by teachers, which influenced the indicators of burnout syndrome and aspects related to the quality of life and health of teachers at different levels of education.

Negative emotions, such as overwork, exhaustion, and frustration, were indicated by several teachers, parallel to the learning during the adaptation process. This duality is well represented in this statement: “We’re all learning and also slowly getting sick.” (T 383, ES).

The study concluded that in the adaptation process, basic education teachers had higher burnout scores than higher education teachers, and teachers who already used digital technologies or worked in distance education had lower burnout scores, which corroborates the literature findings. However, early childhood education stood up as a separate category in the study, leading to individual results. Another contribution of this study was that these findings related to the categories studied specifically for this level of teaching.

Besides this care with early childhood education and the sample, future works could use the same proposed methodology focusing on issues not identified in this article. For instance, this study did not find significant differences regarding gender. However, the literature covers this, and a teacher even expresses: “Domestic work, caring for young children, and the demands imposed by remote work are intense and mainly weaken women researchers, influencing their scientific production and performance, as well as their mental health. Gender inequality has been accentuated by the pandemic.” (T 122, HE).

Future studies could investigate other variables that may have influenced burnout syndrome indicators and aspects related to teachers’ quality of life and health during the pandemic, such as family support.

## Supplementary Information

**Additional file 1.**

## Data Availability

All data generated or analyzed during this study are included in this published article [and its supplementary information files].

## Abbreviations

ANOVA: Analysis of Variance
CTHS: Career and Technical High School
ECE: Early Childhood Education
ES: Elementary School
HE: Higher Education
HS: High School
MBI: Maslach Burnout Inventory
MS: Middle School
T: Teacher
